# Circulating Type I Interferon Levels in the Early Phase of COVID-19 Are Associated With the Development of Respiratory Failure

**DOI:** 10.3389/fimmu.2022.844304

**Published:** 2022-02-14

**Authors:** Kentaro Nagaoka, Hitoshi Kawasuji, Yushi Murai, Makito Kaneda, Akitoshi Ueno, Yuki Miyajima, Yasutaka Fukui, Yoshitomo Morinaga, Yoshihiro Yamamoto

**Affiliations:** ^1^ Department of Clinical Infectious Diseases, Toyama University Graduate School of Medicine and Pharmaceutical Sciences, Toyama, Japan; ^2^ Department of Microbiology, Toyama University Graduate School of Medicine and Pharmaceutical Sciences, Toyama, Japan

**Keywords:** COVID-19, type I interferon, pneumonia, hypoxemia, interleukin-6, CXCL10

## Abstract

**Background:**

The role of type I interferons (IFNs) in the early phase of COVID-19 remains unclear.

**Objectives:**

To evaluate the relationship between IFN-I levels in patients with COVID-19 and clinical presentation, SARS-CoV-2 viral load, and other major pro-inflammatory cytokines.

**Methods:**

This prospective observational study recruited patients hospitalized with COVID-19. The levels of interferon-alpha (IFN-α), interferon-beta (IFN-β), interleukin-6 (IL-6), and C-X-C motif chemokine ligand (CXCL10) within 5 days after symptom onset were measured using an ELISA, in serum from blood collected within 5 days after the onset of symptoms. The SARS-CoV-2 viral load was determined *via* qPCR using nasal-swab specimens and serum.

**Results:**

The study enrolled 50 patients with COVID-19. IFN-α levels were significantly higher in patients who presented with pneumonia or developed hypoxemic respiratory failure (p < 0.001). Furthermore, IFN-α levels were associated with viral load in nasal-swab specimens and RNAemia (p < 0.05). In contrast, there was no significant association between IFN-β levels and the presence of pneumonia or RNAemia, despite showing a stronger association with nasal-swab viral load (p < 0.001). Correlation analysis showed that the serum levels of IFN-α significantly correlated with those of IFN-β, IL-6, and CXCL10, while the levels of IFN-β did not correlate with those of IL-6 or CXCL10.

**Conclusions:**

Serum IFN-I levels in the early phase of SARS-CoV-2 infection were higher in patients who developed hypoxemic respiratory failure. The association between IFN-α, IL-6, and CXCL10 may reflect the systemic immune response against SARS-CoV-2 invasion into pulmonary circulation, which might be an early predictor of respiratory failure due to COVID-19.

## Introduction

Coronavirus disease (COVID-19) is a potentially fatal respiratory infection caused by severe acute respiratory syndrome coronavirus 2 (SARS-CoV-2) (1). Since the first outbreak of COVID-19, this pandemic has negatively affected the capacity of local and regional healthcare systems worldwide, resulting in the temporal exhaustion of in-hospital medical services (2, 3). The necessity of hospitalization due to COVID-19 is largely affected by the presence of respiratory failure, which typically develops several days (at least 3 days) after the onset of the first symptoms (4, 5). Therefore, investigating the initial immune response in the early phase of SARS-CoV-2 infection is important because it may be related to the development of respiratory failure.

Using various integrated approaches (6–9), most studies have compared immune responses between groups of patients with COVID-19 with increasing disease severity. These studies yielded homogenous results; the most severe COVID-19 phenotype was associated with an aggressive inflammatory response with the release of a large amount of pro-inflammatory cytokines, an event known as “cytokine storm”, and described altered cellular immunity, including marked lymphocytopenia and neutrophil-to-lymphocyte ratio (NLR) elevation (10).

Type I interferons (IFNs) have emerged as crucial contributors to the immune response against a SARS-CoV-2 infection (11–13). In humans, the IFN-I family mainly consists of IFN-α and IFN-β (14), which act as inhibitors of viral replication in infected cells and play a defensive role in uninfected cells. The expression of IFN-I is cell-type specific: IFN-α is mainly produced by hematopoietic cells, whereas IFN-β is produced by a broad range of cell types (15). Impairment of IFN-α and increased autoantibodies against IFN-α have been recognized as important contributors to the disease severity in SARS-CoV-2 infection (11–14). In contrast, a recent *in vitro* study reported that the antiviral activity of IFN-β was superior to that of IFN-α against SARS-CoV-2 (16). Nevertheless, the involvement of each IFN-I in the pathogenesis and outcomes of a SARS-CoV-2 infection, particularly the development of hypoxemic respiratory failure, remains unclear.

In this study, we examined the association between pneumonia, hypoxemic respiratory failure, and immune response in the early phase of COVID-19, focusing on circulating IFN-I levels. Moreover, we assessed the levels of other inflammatory cytokines, including IL-6 and CXCL-10, which are known to be initial immune triggers of the cytokine storm in a SARS-CoV-2 infection (17).

The primary outcome of the study was to validate the relationship between SARS-CoV-2 pneumonia, respiratory failure, IFN-α, and IFN-β, and the secondary outcome was to investigate the association between IFN-I and other cytokines.

## Materials and Methods

### Study Design

This study was conducted as part of the Toyama University COVID-19 cohort study; an investigator-initiated, prospective, single-center study that was primarily designed to investigate the clinical, epidemiological, radiological, and microbiological features of COVID-19. In this study, the patients were diagnosed as COVID-19 positive based on quantitative reverse transcription PCR (RT-qPCR) results. Nasal specimens for RT-qPCR were collected and chest computed tomography (CT) were performed at admission. Serum samples were collected and stored at -80°C after each laboratory examination. The study was approved by the Ethical Review Board of the University of Toyama (R2019167), and written informed consent was obtained from all the patients.

The inclusion criteria were as follows: (1) men or women aged 18 years or older; (2) hospitalized at Toyama University Hospital, Toyama, Japan, between April 2021 and June 2021 (during the endemic period in Toyama that was caused by the SARS-CoV-2 B.1.1.7 lineage); (3) positively diagnosed as having a SARS-CoV-2 infection *via* qPCR using nasal-swab specimens; and (4) had the first blood sample collected within 5 days after the onset of symptom. Patients who did not have an initial CT evaluation or whose blood samples were unavailable for subsequent experiments, were excluded from the study.

### Study Participants and Protocol

Data on the patients’ demographics, comorbidities, clinical presentation, laboratory findings, therapy regimen, and prognosis were collected from their medical charts.

When a newly developed inflammatory lesion was detected by a chest CT that was performed on admission, COVID-19 pneumonia was confirmed by trained pulmonary radiologists, KN and YY. This method for diagnosis of pneumonia is consistent with previous reports (18, 19). The patients with no inflammatory lesions were confirmed negative for COVID-19 pneumonia. The chest CT was performed in the supine position during end-inspiration on a multidetector CT scanner by using a slice thickness of 1.0 mm, a high spatial resolution algorithm (SOMATOM Definition AS+; Siemens Healthineers, Forchheim, Germany), and SOMATOM go.Top (Siemens Healthineers).

Hypoxemia requiring oxygen therapy was defined as an SpO2 of ≤93% at rest/motion under room air. This is a universally accepted criterion for the initiation of oxygen therapy in patients with COVID-19 (20).

### Blood Samples

At least 1.0 mL of serum was collected from each patient and divided into three tubes, one of which was used for the cytokine and RNAemia measurements described below. Only serum collected within 5 days after the onset of symptoms was used for the analysis.

In addition, control serum was obtained from healthy immunocompetent volunteers, from Toyama University Hospital. The volunteers were hospital staff who had no known underlying diseases. The blood sampling was conducted under afebrile conditions and the serum was stored and utilized for cytokine measurements. Written informed consent were also obtained from all the volunteers.

### Cytokine Measurement

Serum cytokines and chemokines (IFN-α, IFN-β, IL-6, interleukin-10 (IL-10), and CXCL10) were measured using commercially available ELISA assays, according to the manufacturers’ instructions. The levels of IFN-α, IFN-β, IL-6, IL-10, and CXCL10 were measured using the VeriKine-HS Human IFN Alpha All Subtype ELISA Kit (PBL Assay Science, New Jersey, USA), the VeriKine-HS Human IFN Beta Serum ELISA Kit (PBL Assay Science), the AuthentiKine™ Human IL-6 ELISA Kit (Proteintech, Illinois, USA), the AuthentiKine™ Human IL-10 ELISA Kit (Proteintech), and the Human CXCL10/IP-10 ELISA Kit (Proteintech), respectively. Each sample was measured on first thaw. If an analyte signal was below the background signal, it was set to zero, and if the signal was detectable, but below the manufacturer’s lower limit of quantification, it was set to the lower limit of detection.

### RT-qPCR

RT-qPCR to detect SARS-CoV-2 was performed as previously described (21, 22). Briefly, RNA was extracted from 140 μL of blood serum or supernatant of nasal-swab specimens by using the QIAamp ViralRNA Mini Kit (QIAGEN, Hilden, Germany) or Nippongene Isospin RNA Virus (Nippongene, Tokyo, Japan), respectively according to the manufacturer’s instructions. The viral loads of SARS-CoV-2 were quantified *via* RT-qPCR using a N2-gene-specific primer/probe set according to the Japan National Institute of Infectious Diseases protocol (23). The AcroMetrix COVID-19 RNA Control (Thermo Fisher Scientific, California, USA) was used as a positive control. The detection limit was approximately 0.4 copies/μL (2 copies/5 μL). RNAemia was determined when SARS-CoV-2 was detectable in the blood serum specimens.

### Statistical Analysis

Background factors were expressed as medians (interquartile range) or numbers (percentage). To evaluate intergroup differences, the Wilcoxon test and Fisher’s exact test were used to compare continuous and nominal variables, respectively. For all pairs of immune parameters and viral loads, Spearman’s Rho correlation coefficients were estimated. The results regarding the association between immune parameters are summarized in a correlation matrix. Statistical significance was set at p < 0.05. The statistical program R (version 4.1.018) and GraphPad Prism version 9.0 (GraphPad Software, California, USA) were used for statistical analyses.

## Results

### Study Participants

The clinical characteristics, laboratory findings, treatment, and outcomes of the 50 patients included in this study are summarized in **Table 1**. Age, underlying diseases (none or hypertension), body mass index, and febrile period were significantly different between patients with COVID-19 with and without pneumonia or hypoxemia. No patients with hypoxemic respiratory failure due to COVID-19 required invasive positive pressure ventilation; however, two patients required nasal high-flow oxygen therapy. All patients included in this study survived COVID-19, at least until 30 days after the onset of symptom. None of the patients included in this study had received antiviral medication at the time of blood sampling.

**Table 1:** Clinical feature of patients in the study.

	Total (n=50)	Pneumonia		Developed hypoxemia required oxygen therapy	
		Positive	negative	Positive	Negative
		(n=35)	(n=15)	(n=17)	(n=33)
Age, years	50.0 [34-57]	51 [40-66]^**^	33 [23-50]	58 [53-69] ^††^	39 [26-51]
Sex; male/female	33/17	26/9	7/8	14/3	19/14
Underlying disease					
None	26 (52)	14 (40)^*^	12 (80)	5 (29) ^†^	21 (64)
Hypertension	12 (24)	12 (34) ^*^	0 (0)	8 (47) ^†^	4 (12)
Diabetes mellitus	5 (10)	5 (14)	0 (0)	4 (24) ^†^	1 (3)
Respiratory disease	2 (4)	1 (3)	1 (7)	0 (0)	2 (6)
Body mass index	23.0 [21-26]	24.0 [22-26] ^*^	21.0 [20-23]	24.7 [22-27] ^†^	22.5 [21-25]
Febrile period (days)	4.5 [2-6]	5 [3-6] ^**^	2 [2-4]	6 [6-7] ^††^	3 [2-5]
Treatment					
Remdesivir	18 (36)	18 (51)	0 (0)	17 (100)	1 (3)
Dexamethasone	19 (38)	19 (54)	0 (0)	17 (100)	2 (6)
Heparin	19 (38)	19 (54)	0 (0)	17 (100)	2 (6)
Nasal High Flow	2 (4)	2 (6)	0 (0)	2 (12)	0 (0)
30 days-mortality	0 (0)	0 (0)	0 (0)	0 (0)	0 (0)

Continuous variables are reported as median [interquartile range (IQR) 25-75]. Categorical variables are reported as number (percentages). ^*^; p<0.05, ^**^; p<0.001 vs patients without pneumonia. ^†^; p<0.05, ^††^; p<0.001 vs patients without developing hypoxemia.

### IFN-α and IFN-β Level Analysis

In this study, we performed preliminary experiments to assess the levels of each IFN at different time points in six patients who developed pneumonia (**Supplementary Table 1**). Accordingly, we found that IFN levels significantly decreased 5 days after the initial assessment in five patients. Among them, the levels of IFN-α and IFN-β decreased time-dependently in three patients, even though they developed respiratory failure thereafter. Based on these results, we focused on IFN levels within 5 days of symptom onset, which correspond to the early phase of SARS-CoV-2 infection.

**Figure 1:**
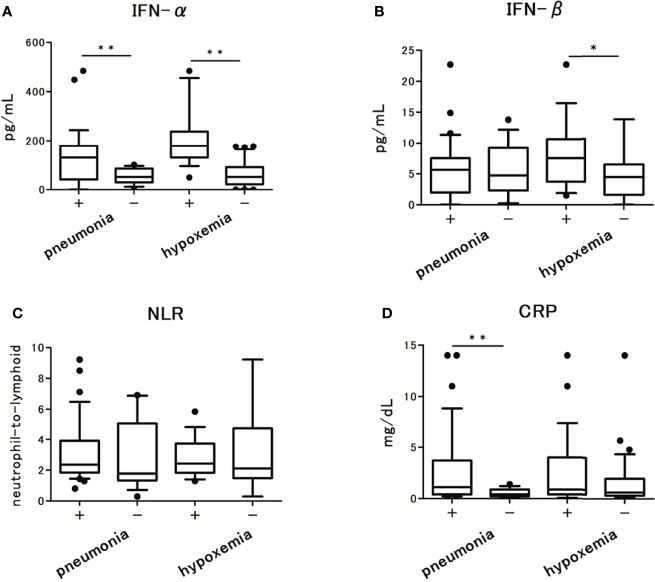
The serum type I Interferon (IFN) levels in patients with COVID-19 and their association with pneumonia and hypoxemic respiratory failure: **(A)** IFN-α levels, **(B)** IFN-β levels. **(C)** CRP levels, and **(D)** Neutrophils-to-lymphocytes ratio in patients with COVID-19. Each level was measured at the time of admission (within 5 days after the onset of symptoms), without hypoxemic respiratory failure at the time. Data are presented as Tukey boxplots and individual values. Nonparametric Mann-Whitney test was used to compare values between groups: ^*^p < 0.05. ^**^p < 0.001.

Next, we assessed the levels of each IFN and cytokine. The results of IFN-α and IFN-β level analyses are summarized in **Figures 1A, B**. The levels of IFN-α were detectable in all patients, but under the lower limit of quantification in three patients. The levels of IFN-β were undetectable in five patients and were below the lower limit of quantification in one patient. In the following analysis, we found that IFN-α levels were significantly higher in patients with pneumonia than in those without pneumonia [130 pg/ml (45-178) vs 51.5 pg/ml (33-85), p < 0.001]. Similarly, IFN-α levels were significantly higher in patients who developed hypoxemia than in those who did not [178 pg/ml [130-236] vs 51.5 pg/ml (23-91), p < 0.001]. In contrast, the levels of IFN-β were not significantly different between patients with and without pneumonia [5.6 pg/ml (2.1-7.5) vs 4.7 pg/ml (3.1-8.2), p = 0.488], but these were significantly higher in patients who developed hypoxemia than in those who did not [7.5 pg/ml (4.5-10) vs 4.5 pg/ml (1.8-6.3), p = 0.008]. The level of C-reactive protein (CRP) and NLR were not significantly different between patients with and without hypoxemia (**Figures 1C, D**).

### Association Between Serum IFN-I Levels and SARS-CoV-2 Viral Load in the Blood/Nasal Swab

**Figure 2:**
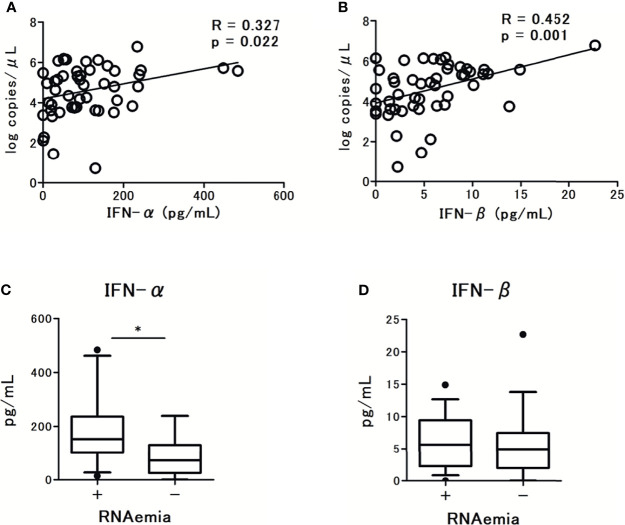
Correlations between serum type I Interferon (IFN-I) levels and SARS-CoV-2 viral load in nasal swab specimens from patients with COVID-19: **(A)** IFN-α levels and **(B)** IFN-β levels. Spearman correlation test was used, and Spearman correlation coefficient is shown. Corresponding logarithmic trendlines are shown. Serum IFN-I levels in patients with COVID-19 and the association with RNAemia, **(C)** IFN-α levels, **(D)** IFN-β levels. Each level was measured at the time of admission (within 5 days after the onset of symptoms). Data are presented as Tukey boxplots and individual values. Nonparametric Mann-Whitney test was used to compare values between groups: ^*^p < 0.05.

To examine the association between the microbiological findings and IFN-I levels, we assessed the viral load in nasal-swab specimens and serum. The levels of IFN-α and IFN-β were significantly associated with SARS-CoV-2 viral load in nasal-swab specimens (r = 0.327; p = 0.327 for IFN-α, and r = 0.452; p = 0.001 for IFN-β; **Figures 2A, B**). Notably, a stronger association was observed with IFN-β than with IFN-α. However, only IFN-α levels were significantly higher in patients with RNAemia than in those without RNAemia (**Figures 2C, D**). The presence of RNAemia was determined by a relatively low viral load [10.4 (4.9-30.0) copies/μL], and the qPCR results were below the detection limit in 35 patients (70% of the study participants). Thus, we analyzed the association between RNAemia and IFN-I levels by the presence of RNAemia.

### Immunoinflammatory Biomarker Level Analysis

**Figure 3:**
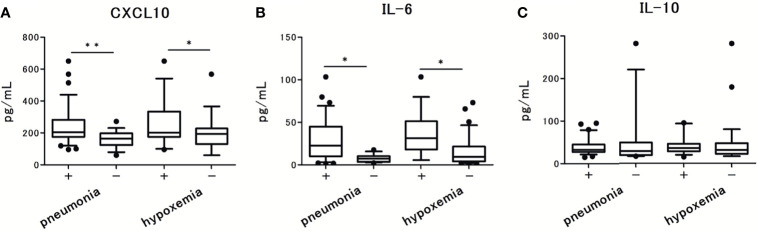
Serum cytokine and chemokine levels in patients with COVID-19 and the associations with pneumonia and hypoxemic respiratory failure: **(A)** CXCL10 levels, **(B)** IL-6 levels, and **(C)** IL-10 levels. Each level was measured at the time of admission (within 5 days after the onset of symptoms), without hypoxemic respiratory failure. One value was excluded from the analyses of IL-6 and IL-10 as an outlier (IL-6 with 476 pg/mL, and IL-10 with 1640 pg/mL). Data are presented as Tukey boxplots and individual values. Nonparametric Mann-Whitney test was used to compare values between groups: ^*^p < 0.05. ^**^p < 0.001.

**Figure 4:**
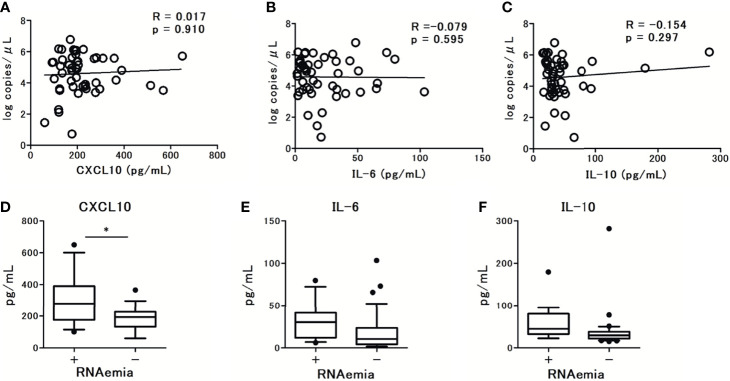
The association observed between serum cytokine levels and SARS-CoV-2 viral load in nasal swab specimens from patients with COVID-19 at admission (within 5 days after the onset of symptoms): **(A)** CXCL10 levels, **(B)** IL-6 levels, and **(C)** IL-10 levels. Spearman correlation test was used, and Spearman correlation coefficient is shown. Corresponding logarithmic trendlines are shown. The association between serum cytokine levels in patients with COVID-19 and RNAemia: **(D)** CXCL10 levels, **(E)** IL-6 levels, and **(F)** IL-10 levels. One value was excluded from the analyses of IL-6 and IL-10 as an outlier (IL-6 with 476 pg/mL, and IL-10 with 1640 pg/mL). Data are presented as Tukey boxplots and individual values. Nonparametric Mann-Whitney test was used to compare values between groups: ^*^p < 0.05.

The levels of CXCL10 and IL-6 were significantly higher in patients with pneumonia and hypoxemia. In contrast, the levels of IL-10 were not significantly different between patients with and without pneumonia or hypoxemia (**Figure 3**). Further analysis revealed that the levels of CXCL10, IL-6, and IL-10 were not associated with the viral load in nasal-swab specimens (**Figures 4A–C**). Moreover, these cytokine levels were not associated with the presence of RNAemia, except for CXCL10 levels (**Figures 4D–F**).

In preliminary experiments, we assessed the levels of tumor necrosis factor (TNF-α), interleukin-17 (IL-17), vascular endothelial growth factor (VEGF), and angiotensin-converting enzyme 2 (ACE-2) by using commercially available ELISA assays, according to the manufacturers’ instructions (**Supplementary Table 1**): TNF-α, IL-17, and VEGF (Chondrex, Washington, USA) and ACE-2 (Abcam, Cambridge, UK). However, the analyte signals of TNF-α, IL-17, and ACE-2 were below the background signals in all patients, and those of VEGF were undetectable in 31 patients (62% of all patients). From these results, we determined that the levels of TNF-α, IL-17, VEGF, and ACE-2 were substantially low in the early phase of SARS-CoV-2 infection; therefore, we excluded these biomarkers from further analysis.

To determine the baseline levels of IFN-I and cytokines, we assessed the inflammatory biomarker levels of IFN-α, IFN-β, IL-6, CXCL10, and IL-10 in healthy volunteers (**Supplementary Table 2**). Amongst, the levels of all biomarkers in healthy volunteers were significantly lower than those in patients with SARS-CoV-2 infection, in particular with IFN-α and IFN-β.

### Correlations Among Immunoinflammatory Biomarker Levels

**Figure 5:**
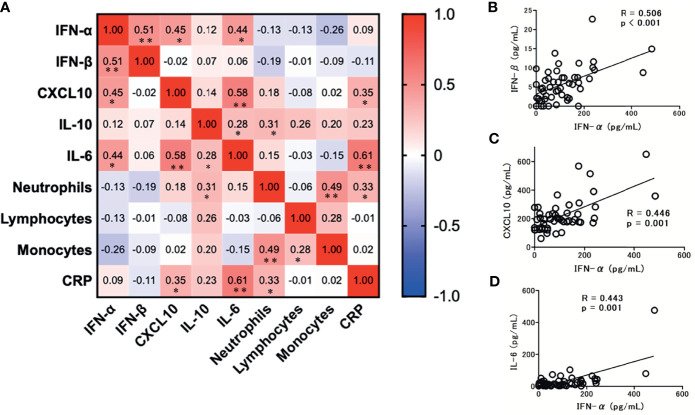
Correlation matrix of immune parameters in patients with COVID-19 at admission (within 5 days after the onset of symptoms). **(A)** Results are presented as a correlation matrix. Spearman correlation coefficients are plotted. Cells were colored according to the strength and trend of correlations (shades of red = positive correlations; shades of blue = negative correlations). ^*^p < 0.05. ^**^p < 0.001. Significant correlations between serum IFN-α levels and IFN/cytokines; **(B)** IFN-β levels, **(C)** CXCL10 levels, and **(D)** IL-6 levels. Spearman correlation test was used, and Spearman correlation coefficient is shown. Corresponding logarithmic trendlines are shown.

Among the tested immunoinflammatory biomarkers, IFN-β (r = 0.51; p < 0.001), CXCL-10 (r = 0.45; p = 0.001), and IL-6 levels (r = 0.44; p = 0.001) were significantly associated with serum IFN-α levels (**Figure 5**). Among the tested inflammatory cytokines, IL-6 levels were strongly associated with CXCL10 (r = 0.58; p < 0.001) and CRP levels (r = 0.61; p < 0.001). However, no significant association was observed between each leukocyte level and the tested immunoinflammatory biomarker levels, except for IL-10 and neutrophil levels (r = 0.31; p = 0.028).

## Discussion

In this study, we demonstrated that the level of serum IFN-α in the early phase of SARS-CoV-2 infection was strongly associated with the presence of pneumonia and the development of hypoxemic respiratory failure. In addition, there was an association between IFN-α levels, the viral load in nasal-swab specimens, and the presence of RNAemia. In contrast, IFN-β levels were not associated with the presence of pneumonia or RNAemia, despite the stronger association observed with nasal-swab viral load. The serum levels of inflammatory cytokines, IL-6 and CXCL10, were significantly associated with pneumonia and hypoxemia, but not with the viral load in nasal-swab specimens. Correlation analysis showed that IFN-α significantly correlated with IFN-β, IL-6, and CXCL10 levels, while IFN-β did not correlate with IL-6 or CXCL10 levels. To our knowledge, this is the first clinical study to reveal the differential expression of IFN-α and IFN-β in the early phase of SARS-CoV-2 infection.

Previous studies suggested that an impaired IFN-I response could be a hallmark of severe COVID-19 (9, 11). However, a recent meta-analysis by Silva et al. (24), which included 15 studies examining the plasma protein levels of IFN-I (α and β), could not confirm a significant association between plasma IFN- I levels and COVID-19 disease severity. In the included studies, IFN-α was measured over 7 days after the onset of symptoms (25–32), and IFN-α levels at the early phase of SARS-CoV-2 infection (within 5 days after the onset of symptoms) were assessed only in one study (30). Galani et al. assessed the IFN-α levels in 32 patients within 5 days after onset using an ELISA kit (Abcam) and found that there was no elevation in the levels of IFN-α in the early phase of SARS-CoV-2 infection. Venet et al. evaluated serum IFN-α levels using a single molecular array in 64 critically ill patients with COVID-19 at a relatively early phase of SARS-CoV-2 infection (serum collected on admission) and compared the IFN-α levels between survivors and non-survivors (33). Although no difference was found between the cohorts, IFN-α levels were the highest in the earlier phase of SARS-CoV-2 infection and decreased time-dependently until 7 days after the initial assessment. To date, the serum levels of IFN-α during the early phase of SARS-CoV-2 infection and their association with disease progression remain unclear. Similarly, the serum levels of IFN-β during the early phase of SARS-CoV-2 infection also remain unclear because the detection of IFN-β was more difficult than that of IFN-α in the previous studies (25, 34).

In this study, we measured IFN-I and cytokine levels within 5 days after the onset of symptoms of a SARS-CoV-2 infection. Notably, our study detected relatively higher levels of IFN-α and IFN-β than those detected in previous studies. As shown by preliminary experiments (**Supplementary Table 1**), the elevation of IFN-I levels in the early phase of infection and their subsequent decrease were observed regardless of disease progression. These results indicate that the temporal elevation of IFN-I levels in the early phase of infection might reflect the initial immune response against SARS-CoV-2, which might appear in a large population of patients with COVID-19. Considering the scarce evidence on IFN-I levels during the early phase of SARS-CoV-2 infection, we believe that our results could extract the crucial timing when IFN-I strongly responds to SARS-CoV-2 infection.

Furthermore, the discrepancy between IFN-α and IFN-β levels during early phase of infection is noteworthy. Our study revealed that IFN-α levels are strongly associated with the presence of pneumonia, RNAemia, and the development of hypoxemic respiratory failure. In contrast, the association between IFN-β and pneumonia or RNAemia was not significant. Serum SARS-CoV-2 RNA viral load, recently termed as RNAemia, reflects the spread of SARS-CoV-2 into circulation, which has been reported as a potential predictor of unfavorable clinical outcomes in patients with COVID-19 (35–37). RNAemia, detected by qPCR, is observed in 1.5% to 50% of patients with a mild-to-severe COVID-19. These results support our findings (RNAemia was detected in 30% of patients with COVID-19). Based on this, we speculate that the discrepancies between IFN-α and IFN-β levels during early phase of infection might be derived from the infected cell types that produce IFN-I (15). Since IFN-α is produced mainly by circulating hematopoietic cells, the elevated IFN-α levels in early phase infection might reflect a systemic immune response against the spread of SARS-CoV-2 into the blood or pulmonary circulation, rather than the local immune response in the nasopharyngeal mucosa. In contrast, the elevated IFN-β levels might reflect the latter immune response, and thus, strongly correlate with the viral load in the nasopharynx. This may explain why there was an association between the levels of IL-6 and CXCL10 with IFN-α levels, but not with those of IFN-β.

**Figure 6:**
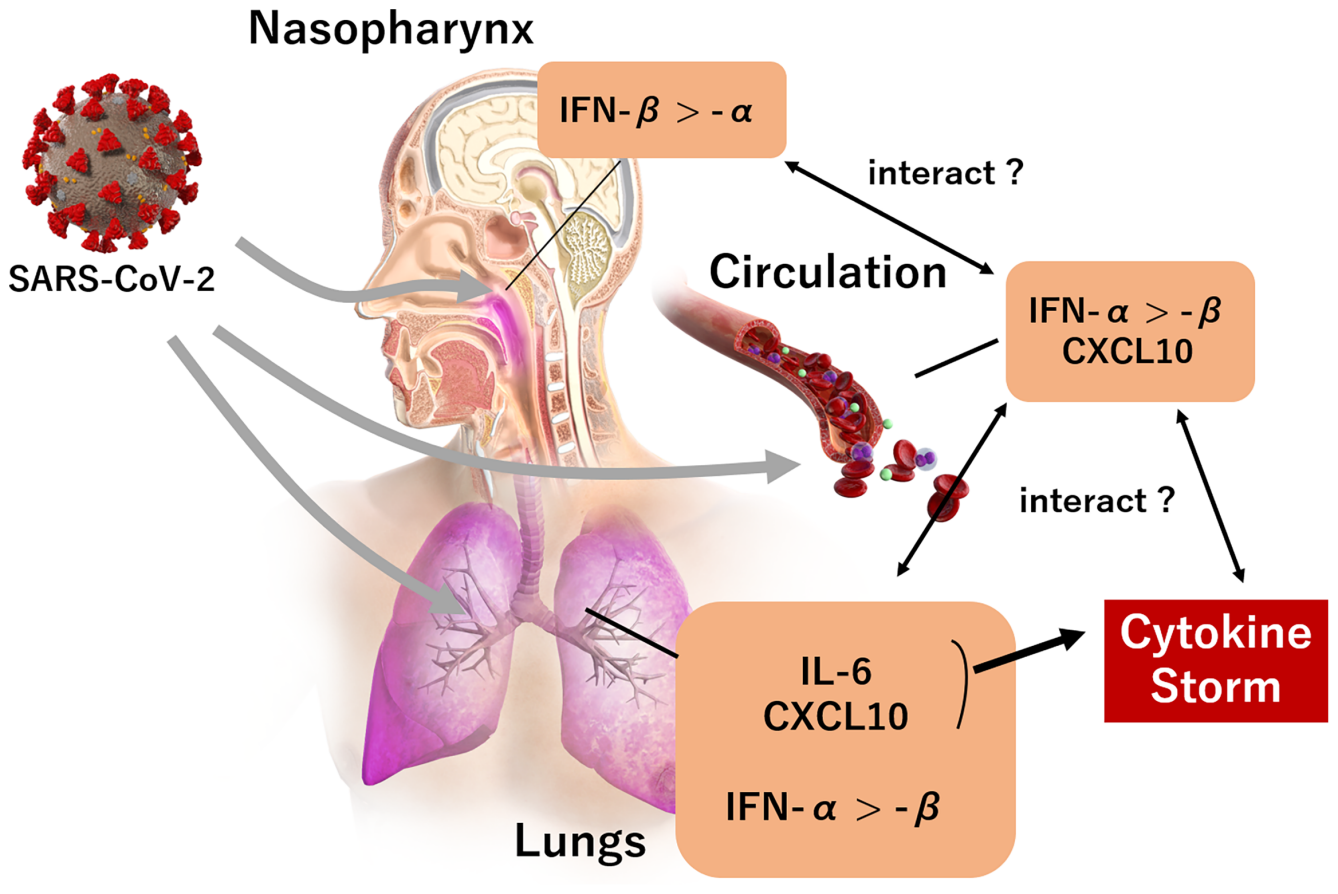
The landscape of IFN-I and predominant cytokines (CXCL10, IL-6) during the early phase of a SARS-CoV-2 infection. A SARS-CoV-2 infection in the nasopharynx induces IFN-β expression rather than IFN-α. IFN-α, IL-6, and CXCL10 expression is induced when the infection reaches the lungs, rather than IFN-β; and the expression of IFN-α and CXCL10 is induced by the presence of SARS-CoV-2 infection in circulation. An increase of IL-6 and CXCL10 in pulmonary circulation subsequently trigger a cytokine storm.

Recent evidence suggests that IL-6 and CXCL10 act as trigger signals of the cytokine storm in COVID-19 (17). A possible mechanism could involve SARS-CoV-2 invasion into the respiratory tract, which in turn stimulates lung epithelial cells to produce cytokines, including IL-6. Thereafter, the secreted IL-6 stimulates several chemokines, including CXCL10, which recruit macrophages from vessels into the interstitium. This creates a cycle involving the overproduction of IL-6 by lung-resident cells as well as by the macrophages recruited by CXCL10. In our study, the blood levels of IL-6 and CXCL10 in the early phase of SARS-CoV-2 infection were significantly associated, and this was compatible with the interactive trigger signals of the cytokine storm. Moreover, IL-6 and CXCL10 levels were significantly associated with the presence of pneumonia and the development of respiratory failure, thereby supporting the hypothesis that the elevation of these inflammatory biomarkers is possibly dominant in the pulmonary circulation. The smaller association between the biomarkers and the viral load in the nasopharynx also supports this notion. Based on these findings, we suggest that significant associations between IFN-α, IL-6, and CXCL10 levels may reflect the systemic immune response, mainly due to the spread of SARS-CoV-2 into pulmonary circulation. A diagram of the proposed mechanism regarding the observed relationship between IFN-I, CXCL10 and IL-6 is shown in **Figure 6**.

The correlation matrix of immune showed that IL-10 was associated with neutrophils, but not with IFN-I. There was no significant association between the amount of circulating leukocyte and IFN-I, IL-6 or CXCL10. These results were not consistent with a previous study which reported an inverse association between lymphocytopenia, IL-10 and IFN-α levels in 54 patients with COVID-19, which included 16 fatal cases (34). IL-10 is an anti-inflammatory cytokine that has pleiotropic roles and that can limit innate immune responses by inhibiting IFN-I (38). Since our study did not include data from fatal cases, the inverse effect of IL-10 on IFN-I could be underestimated. We hypothesize that, at least in the early phase of SARS-CoV-2 infection, IL-10 and circulating leukocytes are not strongly associated with IFN-I.

Aside from IFN-I, type III interferons (IFN-III) have recently received considerable attention as the predominant antiviral cytokines present at the mucosal barriers in the upper respiratory tract of SARS-CoV-2 infected patients (39, 40). Sposito et al. analyzed the pattern and level of expression of IFN-I (α and β), IFN-III (λ1, λ2, λ3) and the transcriptional programs associated with the IFN landscape in the upper or lower respiratory tract of patients with varied severity of COVID-19. They found that high levels of IFN-III, and to a lesser extent IFN-I, characterize the upper airways of patients with high viral burden but reduced disease risk or severity. In contrast, IFN-I were overrepresented in the lower airways of patients with severe COVID-19. These interferons are linked to gene pathways associated with increased apoptosis and decreased proliferation (39). Similarly, Gilbert et al. investigated the mRNA levels of IFN-I and IFN-III in nasopharyngeal swabs from 147 patients with COVID-19, and found that a SARS-CoV-2 infection induced the selective upregulation of IFN-λ1 expression in pediatric patients (≤15 years), whereas the mRNA expression levels of IFN-α, IFN-β, and IFN-λ2/3 was unaffected. Conversely, the infection triggered an upregulation of IFN-α, IFN-β, and IFN-λ2/3 in adults (15-65 years) and the elderly (≥65 years), but there was no modulation of IFN-λ1 expression (40). In these studies, it is not clear at what stage during the SARS-CoV-2 infection the blood samples were taken; therefore, circulating levels of IFN-IIIand its relationship with IFN-I in the early phase of SARS-CoV-2 infection are unclear. Concerning the local immune response, our study demonstrated that IFN-β in the early phase is possibly dominant in the nasopharyngeal mucosa rather than systemic immune response, which is similar to what is observed for IFN-III. However, the exact role and effect of the increased IFN-I levels that are observed during the early phase of SARS-CoV-2 infection are still largely unknown. Further studies are required to examine the interaction of IFN-I with proinflammatory cytokines and IFN-III in the early phase of SARS-CoV-2 infection.

To our knowledge, this is the first study to demonstrate that elevated levels of circulating IFN-I, like those of IL-6 and CXCL10, predict the further development of hypoxemic respiratory failure. Since the anti-viral treatment included in this study was initiated after blood sampling, we believe that the predictive value of IFN-I and cytokines accurately reflect the risk of hypoxemia in the early phase of SARS-CoV-2 infection. The initial elevation of serum IFN-α levels is possibly affected by SARS-CoV-2 systemic invasion, predominantly in the pulmonary circulation. Thus, the detection of IFN-α levels in the early phase of SARS-CoV-2 infection might help identify high-risk patients with respiratory failure who require urgent hospitalization. To date, elevated serum levels of CXCL-10 and IL-6 have been consistently reported in patients with COVID-19, as these are associated with an increased disease severity and risk of mortality (41, 42). We suggest that the establishment of a novel approach focusing on IFN-α and corresponding cytokines in the early phase of SARS-CoV-2 infection would contribute to the early detection of patients with COVID-19 at a high risk of respiratory failure.

The present study has several limitations. First, the single-center observational study design with a relatively small sample size may have result in selection bias. Second, we assessed IFN-I and cytokine levels in serum samples which were not stored at -80°C immediately after drawing (serum was first stored in 4°C, and then transferred to a -80°C freezer). Third, we only examined a single timepoint. Since IFN-I is rapidly and transiently induced by antiviral molecules, the association between serum IFN-I levels and the prognosis of COVID-19 should be further investigated to confirm time-dependent changes. During this study period, patients with mild-to-moderate disease could only stay in hospital for a relatively short term (2-7 days) because of the temporal exhaustion of in-hospital medical services in the region. Thus, the longitudinal assessment of IFN-I activity was difficult in most of the patients. Moreover, IFN-I and cytokine levels in the later phase of SARS-CoV-2 infection would be largely affected by several factors including a variety of treatments and secondary bacterial infection. Future studies are necessary to investigate long-term changes in IFN-I levels, which may minimize those bias. However, this study could include various unvaccinated patients in the same endemic period, which would minimize the bias due to vaccine- or strain-dependent SARS-CoV-2 virulence. Considering the consistent correlation between IFN-I and the major cytokines, we believe that these limitations have not significantly affected the study outcomes.

In conclusion, this study demonstrated that serum IFN-I levels in the early phase of SARS-CoV-2 infection were higher in patients who developed hypoxemic respiratory failure. Analysis of the associations between IFN-I, major inflammatory cytokines, and SARS-CoV-2 viral load revealed that the early elevation of serum IFN-α levels may be affected by SARS-CoV-2 systemic invasion, which could be a predictor of disease progression, including respiratory failure. These findings would encourage further research into the specific role of IFN-I in the early phase of SARS-CoV-2 infection.

## Data Availability Statement

The original contributions presented in the study are included in the article/**Supplementary Material**. Further inquiries can be directed to the corresponding author.

## Ethics Statement

The studies involving human participants were reviewed and approved by The Ethical Review Board of the University of Toyama (R2019167). The patients/participants provided their written informed consent to participate in this study.

## Author Contributions

KN designed and interpreted the clinical data, experimental findings, and prepared the manuscript. KN, with the assistance of YMu, MK, HK, AU, YMi, and YF collected the clinical data and blood. KN and YMo contributed to the analysis of the experimental and microbiological findings. KN, YMu, MK, HK, AU, YMi, YF, and YMo contributed to the analysis of the microbiological findings. KN and YY confirmed the radiological findings. KN and HK confirmed the accuracy of the statistical analysis. All authors contributed to discussions throughout the work. All authors contributed to the article and approved the submitted version.

## Funding

This study was partly supported by the Research Program on Emerging and Re-emerging Infectious Diseases from AMED Grant No. JP20he0622035.

## Conflict of Interest

The authors declare that the research was conducted in the absence of any commercial or financial relationships that could be construed as a potential conflict of interest.

## Publisher’s Note

All claims expressed in this article are solely those of the authors and do not necessarily represent those of their affiliated organizations, or those of the publisher, the editors and the reviewers. Any product that may be evaluated in this article, or claim that may be made by its manufacturer, is not guaranteed or endorsed by the publisher.

## References

[1] Guan WJ Ni ZY Hu Y Liang WH Ou CQ He JX. Clinical Characteristics of Coronavirus Disease 2019 in China. N Eng J Med (2020) 382:1708–20. doi: 10.1056/NEJMoa2002032 PMC7092819 32109013

[2] Ranney ML Griffeth V Jha AK. Critical Supply Shortages - The Need for Ventilators and Personal Protective Equipment During the Covid-19 Pandemic. N Engl J Med (2020) 382:e41. doi: 10.1056/NEJMp2006141 32212516

[3] Mareiniss DP. The Impending Storm: COVID-19, Pandemics and Our Overwhelmed Emergency Departments. Am J Emerg Med (2020) 38:1293–4. doi: 10.1016/j.ajem.2020.03.033 PMC7102611 32253132

[4] Galloway JB Norton S Barker RD Brookes A Carey I Clarke BD. A Clinical Risk Score to Identify Patients With COVID-19 at High Risk of Critical Care Admission or Death: An Observational Cohort Study. J Infect (2020) 81:282–8. doi: 10.1016/j.jinf.2020.05.064 PMC7258846 32479771

[5] Gentilotti E Savoldi A Compri M Górska A De Nardo P Visentin A. Assessment of COVID-19 Progression on Day 5 From Symptoms Onset. BMC Infect Dis (2021) 21:883. doi: 10.1186/s12879-021-06596-5 34454452 PMC8401365

[6] Laing AG Lorenc A Del Molino Del Barrio I Das A Fish M Monin L. A Dynamic COVID-19 Immune Signature Includes Associations With Poor Prognosis. Nat Med (2020) 26:1623–35. doi: 10.1038/s41591-020-1038-6 32807934

[7] Arunachalam PS Wimmers F Mok CKP Perera R Scott M Hagan T. Systems Biological Assessment of Immunity to Mild Versus Severe COVID-19 Infection in Humans. Science (2020) 369:1210–20. doi: 10.1126/science.abc6261 PMC7665312 32788292

[8] Silvin A Chapuis N Dunsmore G Goubet AG Dubuisson A Derosa L. Elevated Calprotectin and Abnormal Myeloid Cell Subsets Discriminate Severe From Mild COVID-19. Cell (2020) 182:1401–18. doi: 10.1016/j.cell.2020.08.002 PMC7405878 32810439

[9] Schulte-Schrepping J Reusch N Paclik D Bassler K Schlickeiser S Zhang B. Severe COVID-19 Is Marked by a Dysregulated Myeloid Cell Compartment. Cell (2020) 182:1419–40. doi: 10.1016/j.cell.2020.08.001 PMC7405822 32810438

[10] Li X Liu C Mao Z Xiao M Wang L Qi S. Predictive Values of Neutrophil-to-Lymphocyte Ratio on Disease Severity and Mortality in COVID-19 Patients: A Systematic Review and Meta-Analysis. Crit Care (2020) 24:647. doi: 10.1186/s13054-020-03374-8 33198786 PMC7667659

[11] Hadjadj J Yatim N Barnabei L Corneau A Boussier J Smith N. Impaired Type I Interferon Activity and Infammatory Responses in Severe COVID-19 Patients. Science (2020) 369:718–24. doi: 10.1126/science.abc6027 PMC7402632 32661059

[12] Bastard P Rosen LB Zhang Q Michailidis E Hofmann HH Zhang Y. Autoantibodies Against Type I IFNs in Patients With Life-Threatening COVID-19. Science (2020) 370:eabd4585. doi: 10.1126/science.abd4585 32972996 PMC7857397

[13] Acharya D Liu G Gack MU. Dysregulation of Type I Interferon Responses in COVID-19. Nat Rev Immunol (2020) 20:397–8. doi: 10.1038/s41577-020-0346-x PMC7249038 32457522

[14] Trouillet-Assant S Viel S Gaymard A Pons S Richard JC Perret M. Type I IFN Immunoprofling in COVID-19 Patients. J Allergy Clin Immunol (2020) 146:206–8. doi: 10.1016/j.jaci.2020.04.029 PMC7189845 32360285

[15] Crouse J Kalinke U Oxenius A. Regulation of Antiviral T Cell Responses by Type I Interferons. Nat Rev Immunol (2015) 15:231–42. doi: 10.1038/nri3806 25790790

[16] Mantlo E Bukreyeva N Maruyama J Paessler S Huang C. Antiviral Activities of Type I Interferons to SARS-CoV-2 Infection. Antiviral Res (2020) 179:104811. doi: 10.1016/j.antiviral.2020.104811 32360182 PMC7188648

[17] Coperchini F Chiovato L Rotondi M. Interleukin-6, CXCL10 and Infiltrating Macrophages in COVID-19-Related Cytokine Storm: Not One for All But All for One! Front Immunol (2021) 12:668507. doi: 10.3389/fimmu.2021.668507 33981314 PMC8107352

[18] Prokop M Everdingen W Vellinga TR Ufford HQ Stöger L Beenen L. CORADS: A Categorical CT Assessment Scheme for Patients Suspected of Having COVID-19—Definition and Evaluation. Radiology (2020) 296:E97–104. doi: 10.1148/radiol.2020201473 32339082 PMC7233402

[19] Machnicki S Patel D Singh A Talwar A Mina B Oks M. The Usefulness of Chest CT Imaging in Patients With Suspected or Diagnosed COVID-19: A Review of Literature. Chest (2021) 160:652–70. doi: 10.1016/j.chest.2021.04.004 PMC8056836 33861993

[20] Wang YC Lu MC Yang SF Bien MY Chen YF Li YT. Respiratory Care for the Critical Patients With 2019 Novel Coronavirus. Respir Med (2021) 186:106516. doi: 10.1016/j.rmed.2021.106516 34218168 PMC8215880

[21] Kawasuji H Takegoshi Y Kaneda M Ueno A Miyajima Y Kawago K. Transmissibility of COVID-19 Depends on the Viral Load Around Onset in Adult and Symptomatic Patients. PloS One (2020) 15:e0243597. doi: 10.1371/journal.pone.0243597 33296437 PMC7725311

[22] Kawasuji H Morinaga Y Tani H Yoshida Y Takegoshi Y Kaneda M. SARS-CoV-2 RNAemia With Higher Nasopharyngeal Viral Load Is Strongly Associated With Severity and Mortality in Patients With COVID-19. J Med Virol (2022) 94:147–53. doi: 10.1002/jmv.27282 PMC8426802 34411312

[23] Shirato K Nao N Katano H Takayama I Saito S Kato F. Development of Genetic Diagnostic Methods for Novel Coronavirus 2019 (Ncov-2019) in Japan. Jpn J Infect Dis (2020) 73:304–7. doi: 10.7883/yoken.JJID.2020.061 32074516

[24] da Silva RP Gonçalves JIB Zanin RF Schuch FB de Souza APD. Circulating Type I Interferon Levels and COVID-19 Severity: A Systematic Review and Meta-Analysis. Front Immunol (2021) 12:657363. doi: 10.3389/fimmu.2021.657363 34054820 PMC8149905

[25] Saínchez-Cerrillo I Landete P Aldave B Saínchez-Alonso S Saínchez-Azofra A Marcos-Jimeínez A. Covid-19 Severity Associates With Pulmonary Redistribution of CD1c+ DC and Inflammatory Transitional and Nonclassical Monocytes. J Clin Invest (2020) 130:6290–300. doi: 10.1172/JCI140335 PMC7685723 32784290

[26] Liu Y Zhang C Huang F Yang Y Wang F Yuan J. Elevated Plasma Levels of Selective Cytokines in COVID-19 Patients Reflect Viral Load and Lung Injury. Natl Sci Rev (2020) 7:6. doi: 10.1093/nsr/nwaa037 PMC7107806 34676126

[27] Henry BM Benoit S Vikse J Berger B Pulvino C Hoehn J. The AntiInflammatory Cytokine Response Characterized by Elevated Interleukin-10 is a Stronger Predictor of Severe Disease and Poor Outcomes Than the Pro-Inflammatory Cytokine Response in Coronavirus Disease 2019 (COVID-19). Clin Chem Lab Med (2020) 59:599–607. doi: 10.1515/cclm-2020-1284 33554561

[28] Lucas C Wong P Klein J Castro TBR Silva J Sundaram M. Longitudinal Analyses Reveal Immunological Misfiring in Severe Covid19. Nature (2020) 584:463–9. doi: 10.1038/s41586-020-2588-y PMC7477538 32717743

[29] Chi Y Ge Y Wu B Zhang W Wu T Wen T. Serum Cytokine and Chemokine Profile in Relation to the Severity of Coronavirus Disease 2019 in China. J Infect Dis (2020) 222:746–54. doi: 10.1093/infdis/jiaa363 PMC7337752 32563194

[30] Thwaites RS Uruchurtu AS Siggins MK Liew F Russell CD Moore SC. Inflammatory Profiles Across the Spectrum of Disease Reveal a Distinct Role for GM-CSF in Severe COVID-19. Sci Immunol (2021) 6:eabg9873. doi: 10.1126/sciimmunol.abg9873 33692097 PMC8128298

[31] Tincati C Cannizzo ES Giacomelli M Badolato R Monforte A Marchetti G. Heightened Circulating Interferon-Inducible Chemokines, and Activated ProCytolytic Th1-Cell Phenotype Features Covid-19 Aggravation in the Second Week of Illness. Front Immunol (2020) 11:1–12. doi: 10.3389/fimmu.2020.580987 33193384 PMC7606391

[32] Galani IE Rovina N Lampropoulou V Triantafyllia V Manioudaki M Pavlos E. Untuned Antiviral Immunity in COVID-19 Revealed by Temporal Type IIII Interferon Patterns and Flu Comparison. Nat Immunol (2021) 22:32–40. doi: 10.1038/s41590-020-00840-x 33277638

[33] Venet F Cour M Rimmelé T Viel S Yonis H Coudereau R. Longitudinal Assessment of IFN-I Activity and Immune Profile in Critically Ill COVID-19 Patients With Acute Respiratory Distress Syndrome. Crit Care (2021) 25:140. doi: 10.1186/s13054-021-03558-w 33845874 PMC8040759

[34] Contoli M Papi A Tomassetti L Rizzo P Vieceli Dalla Sega F Fortini F. Blood Interferon-α Levels and Severity, Outcomes, and Inflammatory Profiles in Hospitalized COVID-19 Patients. Front Immunol (2021) 12:648004. doi: 10.3389/fimmu.2021.648004 33767713 PMC7985458

[35] Miki S Sasaki H Horiuchi H Miyata N Yoshimura Y Miyazaki K. On-Admission SARS-CoV-2 RNAemia as a Single Potent Predictive Marker of Critical Condition Development and Mortality in COVID-19. PloS One (2021) 16:e0254640. doi: 10.1371/journal.pone.0254640 34255796 PMC8277033

[36] Ram-Mohan N Kim D Zudock EJ Hashemi MM Tjandra KC Rogers AJ. SARS-CoV-2 RNAemia Predicts Clinical Deterioration and Extrapulmonary Complications From COVID-19. Clin Infect Dis (2021) 74:218–26. doi: 10.1101/2020.12.19.20248561 PMC8135992 33949665

[37] van Riel D Embregts CWE Sips GJ van den Akker JPC Endeman H van Nood E. Temporal Kinetics of RNAemia and Associated Systemic Cytokines in Hospitalized COVID-19 Patients. mSphere (2021) 6:e0031121. doi: 10.1128/mSphere.00311-21 34047654 PMC8265646

[38] Ito S Ansari P Sakatsume M Dickensheets H Vazquez N Donnelly RP. Interleukin-10 Inhibits Expression of Both Interferon Alpha- and Interferon Gamma- Induced Genes by Suppressing Tyrosine Phosphorylation of STAT1. Blood (1999) 93:1456–63. doi: 10.1182/blood.V93.5.1456.404a34_1456_1463 10029571

[39] Sposito B Broggi A Pandolfi L Crotta S Clementi N Ferrarese R. The Interferon Landscape Along the Respiratory Tract Impacts the Severity of COVID-19. Cell (2021) 184:4953–68.e16. doi: 10.1016/j.cell.2021.08.016 34492226 PMC8373821

[40] Gilbert C Lefeuvre C Preisser L Pivert A Soleti R Blanchard S. Age-Related Expression of IFN-λ1 Versus IFN-I and Beta-Defensins in the Nasopharynx of SARS-CoV-2-Infected Individuals. Front Immunol (2021) 12:750279. doi: 10.3389/fimmu.2021.750279 34858406 PMC8631500

[41] Lorè NI De Lorenzo R Rancoita PMV Cugnata F Agresti A Benedetti F. CXCL10 Levels at Hospital Admission Predict COVID-19 Outcome: Hierarchical Assessment of 53 Putative Inflammatory Biomarkers in an Observational Study. Mol Med (2021) 27:129. doi: 10.1186/s10020-021-00390-4 34663207 PMC8521494

[42] Shcherbak SG Anisenkova AY Mosenko SV Glotov OS Chernov AN Apalko SV. Basic Predictive Risk Factors for Cytokine Storms in COVID-19 Patients. Front Immunol (2021) 12:745515. doi: 10.3389/fimmu.2021.745515 34858403 PMC8631447

