# Supplementary material for: Circulating Type I Interferon Levels in the Early Phase of COVID-19 Are Associated With the Development of Respiratory Failure
Source: Front Immunol. 2022 Feb 14;13:844304. doi: 10.3389/fimmu.2022.844304 (PMC8882823; doi:10.3389/fimmu.2022.844304)
Supplement: Supplementary file 1 [file Table_1.pdf]

Supple Table 1 Time-dependent change in inflammatory biomarker in 6 patients with COVID-19.

(pg/mL)	day 0-5	5 days after
IFN-α	230.6 ± 76.2	20.5 ± 18.5 *
IFN-β	14.1 ± 3.7	2.4 ± 0.8 *
CXCL10	16.7 ± 3.5	10.6 ± 2.0
IL-6	61.4 ± 35.7	16.2 ± 4.6
IL-10	22.7 ± 6.0	21.5 ± 3.6
VEGF	35.8 ± 25.9	114 ± 63.4
TNF-α	0	0
IL-17	0	0
ACE-2	0	0

Data are expressed as the mean and SEM. *; $p<0.05$ vs base line.
