# Supplementary material for: Circulating Type I Interferon Levels in the Early Phase of COVID-19 Are Associated With the Development of Respiratory Failure
Source: Front Immunol. 2022 Feb 14;13:844304. doi: 10.3389/fimmu.2022.844304 (PMC8882823; doi:10.3389/fimmu.2022.844304)
Supplement: Supplementary file 2 [file Table_2.pdf]

Supple Table 2 Inflammatory biomarkers in healthy volunteers.

	Healthy volunteer (n=25)	COVID-19 in this study (n=50)
Age, years	36 [31-55]	50 [34-57]
Male/Female	17/8	33/19
IFN-α (pg/mL)	0 [0-0.33]	88.5 [36-158]**
IFN-β (pg/mL)	0 [0-0]	5.29 [2.2-7.5]**
CXCL10 (pg/mL)	82.2 [27-114]	194.7 [144-237]**
IL-6 (pg/mL)	3.82 [2.6-6.1]	14.5 [6.5-33]*
IL-10 (pg/mL)	7.86 [3.5-12]	32.9 [24-46]*

Continuous variables are reported as median [interquartile range (IQR) 25-75].
*; $p<0.05$. **; $p<0.001$ vs healthy volunteers.
